# Biomarkers in skin autoimmunity—An update on localised scleroderma

**DOI:** 10.1002/ski2.335

**Published:** 2024-01-12

**Authors:** Adrián Hernández‐Bustos, Begona Bolos, Kira Astakhova

**Affiliations:** ^1^ Department of Chemistry Technical University of Denmark Kongens Lyngby Denmark

## Abstract

Human autoimmune diseases are complex and highly diverse conditions that can be of localised or systemic nature. Understanding the basic biology of autoimmune diseases goes hand in hand with providing the clinics with valuable biomarkers for managing these diseases. The focus of this review is paid to localised scleroderma, an autoimmune disease affecting skin and subcutaneous tissue. Localised scleroderma has very few serological biomarkers for clinical analyses distinguishing it from main differentials, and yet noneffective prognostic biomarkers. With this regard, the review covers well‐established and new biomarkers such as cell surface proteins, autoantibodies and cytokines. In recent few years, several new biomarkers have been suggested, many provided with modern genomic studies. This includes epigenetic regulation of DNA, RNA transcriptomics and regulatory RNA such as microRNA and long non‐coding RNA. These findings can for the first time shed light on the genetic mechanisms behind the disease and contribute to improved diagnosis and treatment.

## Graphical abstract

Newest research on biomarkers in localised scleroderma reviewed.

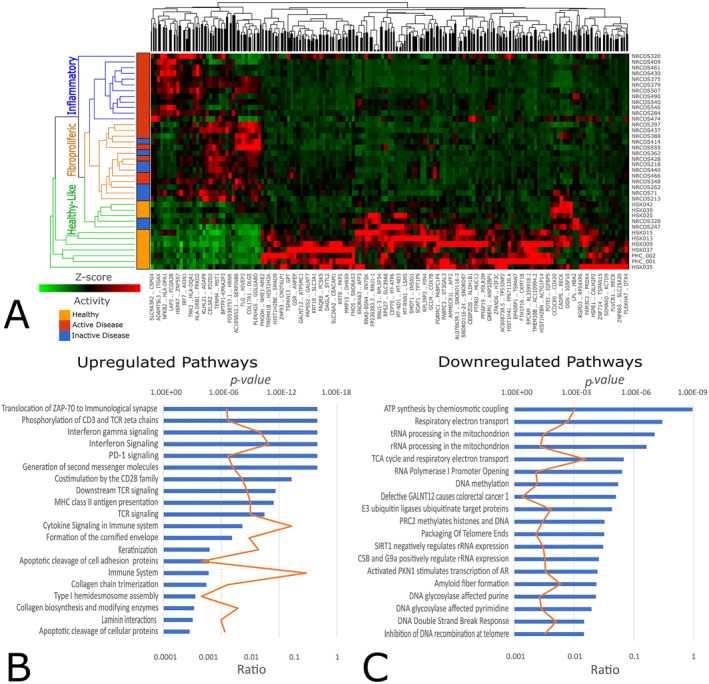

**What is already known?**

- Localised scleroderma (LS) is an autoimmune disease affecting skin and subcutaneous tissue
- LS has very few serological biomarkers for clinical analyses distinguishing it from main differentials, and yet noneffective prognostic biomarkers.

**What does this study add?**

- This review covers well‐established and new biomarkers such as cell surface proteins, autoantibodies and cytokines
- New promising biomarkers include epigenetic markers, regulatory RNA and regulatory proteins

## 1 INTRODUCTION

Mechanisms of autoimmune diseases are still not fully understood, inspiring the research community to study them with continuously advanced experimental and clinical approaches. Thanks to these efforts it is now confirmed that multiple autoimmune diseases are caused by a prolonged or chronic inflammatory response.^1^, ^2^ These responses have origin in genetic predisposition and epigenetic factors.^1^ Such precise biomarkers could be used for tracking diseases and potentially, guide treatment. Besides diagnostic and treatment follow up purposes, monitoring common biomarkers could reveal biological details on different autoimmune diseases and their interconnection.

Localized scleroderma (LS), or morphea, is an autoimmune disease typically leading to skin inflammation and thickness distributed in various parts of the body, as well as affecting the extracutaneous tissues. Localised scleroderma can be classified based on the extent and degree of fibrosis into different groups. Traditionally, it has been classified into subtypes plaque, generalised, bullous, linear, and deep.^3^ However, according to the Padua consensus, it can be divided in the following groups: circumscribed (including subtypes superficial and deep), linear (including the subtypes trunk and head), generalised, panclerotic and mixed,^4^ being this the most accepted classification.^5^ While its pathogenesis is not fully known, factors such as genetic predisposition and epigenetics,^6^, ^7^, ^8^ vascular dysregulation or Th1/Th2/Th17 imbalance through the different stages^9^ lead to the activation of inflammatory and profibrotic pathways that ends into an upregulation on collagen deposition.

With a reported incidence of 3.4–27 per 100.000,^10^ or 4–27 new cases per million children per year reported in,^9^ LS affects mainly women and one third of the patients are paediatric.^10^ The treatment is mainly based on methotrexate with efficacy 67% in paediatric patients.^11^, ^12^, ^13^ Corticosteroids are applied as well. However, most patients relapse after corticosteroids' discontinuation.^14^

Localised scleroderma and systemic sclerosis (Ssc) share skin histopathologic changes, reason why sometimes certain subtypes of LS as pansclerotic LS can be confounded with SSc.^15^ However, these two diseases differ in the distribution and pattern of skin involvement. The associated extracutaneous and internal organ manifestations are mild in LS,^16^ and are more severe and common in SSc.^17^ Remarkably, LS cannot evolve into SSc. Immunogenetically, human leukocytes antigens (HLA) type II genes are upregulated in LS, being type II specific upregulation, while in SSc it is type I upregulation that takes place.^18^ As it will be further discussed, both diseases share the presence of certain pathogenic autoantibodies, nevertheless, are genetically different.

Protein biomarkers for LS have been far less successful compared to other autoimmune diseases such as lupus or rheumatoid arthritis (RA). Since there is no serological marker specific for the disease or characteristic parameters in LS patients,^19^ diagnostics are based on clinical observations and, in cases where the LS type is not evident or the inflammatory state has to be defined, skin biopsies are used.^20^, ^21^ Both serological and tissue samples are used to monitor LS. Other methods are based on imaging, using techniques as ultrasonography,^22^, ^23^ infrared thermography,^13^, ^24^ and optical coherence tomography.^25^ Nevertheless, misdiagnose^26^ and risk of extracutaneous involvement which commonly associates with morbidity in LS,^27^ require fast and robust methods to diagnose and monitor LS and its subtypes.

As to the genetic signature of LS, specific HLA class I and class II alleles are associated with generalised and linear subtypes. Interestingly, LS is immunogenetically distinct from other scleroderma types.^6^ However, risk alleles in morphea are also associated with conditions such as RA and other autoimmune conditions.^6^ The role of HLA products in regulating interactions of immune cells is well known,^28^ and therefore, the specific HLA profile of morphea could lead to B cells producing certain cytokines and autoantibodies contributing to disease progression.^6^, ^29^

The main objective of this review is to give an update on relevant biomarkers for diagnosing and studying LS. In addition to previously reviewed biomarkers,^30^ we also include genomic studies, cell surface and endothelial biomarkers, and give a brief overview of upcoming clinical trials that aim at genetic studies of LS.

## 2 BIOMARKERS IN LOCALISED SCLERODERMA

In the chapter below we sum up recent advances in biomarkers in scleroderma and classify them according to clinical relevance as follows: biomarkers of disease activity, biomarkers that enable distinguishing LS from the main differentials, and prognostic indicators including those that can monitor response to treatment. Both clinically established biomarkers and those in the research stage are included, to show the ongoing development in the field and to make the clinicians aware of the upcoming new tests.

### 2.1 Biomarkers of disease activity and extracutaneous involvement

Cell surface and endothelial biomarkers can be evaluated using skin biopsy and reveal disease activity and complications (Table 1). Cluster of differentiation CD34 is a biomarker used to identify certain cell populations, particularly hematopoietic stem and progenitor cells, endothelial progenitor cells, and various types of mesenchymal cells. CD34 is a cell surface glycoprotein expressed on endothelial cells, including those present in blood vessels. Studies have shown decreased expression of CD34 on endothelial cells in LS lesions.^31^, ^32^

**TABLE 1:** Overview of biomarkers used in Localised scleroderma (LS) diagnosis and studies for assessing LS activity and complications (2000–2022).^a^

Biomarker	Tissue (T) or serum (S) sample	Activity (A) and/or complications (C) association	Cohort size (year)	Biological association	Details	(Refs.)
CD34	T	A	20 keloid,	Cell surface glycoprotein expressed on endothelial cells.	Decreased expression of CD34 on endothelial cells in localised scleroderma lesions. CD34 can also be used to differentiate from other fibrotic diseases or to follow treatment response.	31, 32
			20 lacaziosis			
			20 LS patients (2022)			
FXIIIa	T	A	30 LS patients (2013)	Transglutaminase related to blood clots stabilisation by fibrin cross‐linking.	FXIIIa + dendritic cells can provide insights into the immune and inflammatory processes occurring in the affected skin.	32
CD1a	T	A	6 LS patients	Cell surface protein in immune Langerhans dendritic cells. These are related to immune responses and antigen presentation.	They can be used to identify and study Langerhans cells in the skin and are associated with disease onset and progression.	33
			3 HC (2008)			
CD86	T	A	6 LS patients	Cell surface protein primarily associated with antigen‐presenting cells. It plays a role on immune responses, T‐cell activation and regulation.	Increased expression of CD86 can indicate immune activation and inflammation in the affected skin of individuals with LS.	34
			3 HC (2008)			
sCD30	S	A	55 LS patients	Circulating form of CD30, a cell surface protein primarily expressed on activated T cells.	Potential biomarker to assess immune system activity and inflammation, as well as disease progression or response to treatment.	35
			15 SSc patients			
			20 HC (2000)			
RF	S	A/C	43 LS patients	RFs are antibodies that target the Fc portion of IgGs as immune response regulators.	RF was present in 30% of LS patients. Patients with generalised morphea can be differentiated by the dysregulated isotype of RF.	36
			14 HC (2005)			
ANAs	S/T	A/C	750 LS patients^a^ (2005)	In LS, autoreactive T and B cells produce pathogenic antibodies such ANAs, AHAs or anti‐ssDNA autoantibodies that attack healthy tissues.	Present in patients with autoimmune connective tissue disease. ANAs were found in the serum of 36%–57.9% of LS patients (depends on the cohort).	37, 38, 39, 40, 41, 42
					It is associated to risk of relapse and extracutaneous involvement.	
anti‐ssDNA autoantibodies	S	A/C	69 LS patients		AHAs were found in 32%–39% of the LS patients, and anti‐ssDNA in 29%–30% of the LS patients, correlating to disease severity.	43
AHAs	S	A/C	71 HC (2008)		Both are commonly proposed as markers for higher risk of muscle and joint morbidity	
Vimentin p16	S/T	A	20 keloid, 20 lacaziosis 20 LS patients (2022)	Vimentin has been suggested to be the main myofibroblast general marker of the fibrotic process. p16 is a tumour suppressor protein.	Vimentin, p16 and FOXP3 were overexpressed in the affected skin for all three fibrotic pathologies.	31
FOXP3				FOXP3 suppresses expression of many genes including IL‐2 and effector T‐cell cytokines.		
IL‐2	S	A	73 LS patients 26 HC (2016)	Dysregulated production of cytokines has a critical role in autoimmune diseases and LS.	All the cytokines were increased and related to disease activity.	44
IL‐2R	S					
IL‐12	S					
CXCL9	S					
CXCL10	S					
CCL18	S	A	74 LS patients 22 HC (2019)		CCL18 allowed to differentiate between active and inactive disease.	45
sVCAM	S	A		sVCAM is related to the transport of the leukocytes to the area where inflammation takes place.	These markers were found upregulated in the serum of LS patients	
Gal‐9	S	A		Gal‐9 is a *β*‐galactoside binding lectin known for its immunomodulatory role.		
TIE‐1	S	A		TIE‐1 has a role in the regulation of lymphatic and cardiovascular development.		
SPARC	S	A	15 LS patients	Fibrosis mediator present only at sites of tissue remodelling and wound repair for different diseases.	SPARC was upregulated in patients with all types of sclerosis, especially elevated in those with LS.	46
			78 Sclerotic patients			
			15 HC (2017)			
MBP antibodies	S	A	70 LS patients	In LS, autoreactive T and B cells produce pathogenic antibodies that attack healthy tissues.	MBP was found in 71.4% of a cohort of 50 patients, differing significantly from HCs, SSc patients and correlating to higher disease activity.	47, 48
			30 SSc patients			
			35 HC (2022)			

^a^ AHA, Antihistone antibodies; ANA, antinuclear antibodies; anti‐ssDNA, anti‐single strand DNA; CD, Cluster Differentiation; FOXP3, forkhead box P3; FXIIIa, Coagulation factor XIIIa; Gal‐9, Galectin‐9; HC, healthy control; IL, interleukin; LS, localized scleroderma; MBP, Myelin Basic Protein; RF, Rheumatoid Factor; sCD, soluble Cluster Differentiation; SPARC, Secreted Protein Acidic and Rich in Cysteine; SSc, Systemic Sclerosis; sVCAM, soluble vascular cell adhesion molecule; TIE‐1, Tyrosine‐protein kinase receptor.

Factor XIIIa (FXIIIa) is a biomarker that is commonly used to identify and characterise certain cells within the skin, particularly dendritic cells known as dermal dendrocytes or FXIIIa + dendritic cells. These cells are a type of antigen‐presenting cell found in the skin and play a role in wound healing, tissue repair, and immune regulation. In the context of LS, FXIIIa + dendritic cells can provide insights into the immune and inflammatory processes occurring in the affected skin.^32^

CD1a is a cell surface protein that serves as a biomarker for a specific type of immune cell known as Langerhans cells. Langerhans cells are dendritic cells present in the skin and mucous membranes, and they play a significant role in immune responses and antigen presentation. In LS, CD1a is used as a biomarker to identify and study Langerhans cells in the skin associated with disease onset and progression.^33^

CD86, also known as B7‐2, is a cell surface protein primarily associated with antigen‐presenting cells, particularly dendritic cells, macrophages, and B cells. CD86 plays a critical role in immune responses, including T‐cell activation and regulation. In the context of LS, CD86 is relevant for monitoring the immune response and inflammatory processes. Increased expression of CD86 can indicate immune activation and inflammation in the affected skin of individuals with LS.^34^

Soluble CD30 (sCD30) is a circulating form of CD30, a cell surface protein and member of the tumour necrosis factor receptor superfamily. CD30 is primarily expressed on activated T cells, including Th2 cells and T‐regulatory cells, and is involved in immune responses. In the context of LS, serum levels of sCD30 has been investigated as a potential biomarker to assess immune system activity and inflammation.^35^ sCD30 is associated with Th2‐type immune responses, which are involved in allergic and inflammatory conditions. Changes in sCD30 levels could be indicative of disease progression or response to treatment.

All these biomarkers are studied in skin biopsies with immunohistochemistry methods. Alternatively, the cell types can be detected in blood with cell sorting techniques.

As to serological tests, they are not common for the diagnosis of LS, even not recommended in guidelines.^20^, ^49^ However, serological biomarkers such as aldolase, creatinine phosphokinase, lactate dehydrogenase, C‐reactive protein and rheumatoid factor (RF) are commonly measured (Table 1) and associate with the LS activity.^20^, ^37^, ^50^ RF was present in 30% of a cohort of 43 LS patients and 14 controls, especially for patients with generalised morphea which can be differentiated by the dysregulated isotype of RF.^36^

The opinion on relevance of ANAs, extractable nuclear antigen antibodies and antibodies to single stranded DNA (anti‐ssDNA) is conflicted since a big percentage of LS patients (50% or more, depending on the cohort and the subtype of LS) is negative on them.^20^, ^49^ In different cohorts, the biggest of them being 671 LS patients,^37^ ANAs were only found in the serum of 36%–57.9% of them.^37^, ^38^, ^39^, ^40^, ^41^, ^42^ In addition, antinuclear antibodies (ANA) positivity combined with older LS onset age is thought to be a potential marker for risk of relapse.^41^ Besides, ANA positivity has been associated with extracutaneous involvement.^16^ This is the case in the study by Li et al. who found extracutaneous involvement associated with more medication use, longer treatment durations, and greater disease burden.^16^

Anti‐histone antibodies (AHAs) were found in 32%–39% of the LS patients, and anti‐ssDNA in 29%–30% of the LS patients.^42^, ^43^ A cohort of 187 LS patients found AHAs and anti‐ssDNA antibodies in only 12% and 8% of the patients, respectively.^51^ Despite the dependence of the autoantibodies levels on the cohort and the low percentage of patients that are positive on them, some authors propose the use of them combined as a marker for higher risk of muscle and joint morbidity.^52^

A recent study showed that myelin basic protein (MBP) antibodies measured in serum of the 27.3% of a cohort of 139 LS patients correlate to higher disease activity.^47^ Smaller cohort of 50 LS patients found these antibodies in 71.4% of the patients.^48^

Along with autoantibodies, cytokines have a unique signature in LS.^8^, ^42^ Thus, serum levels of a cohort consisting of 73 LS patients and 26 healthy controls proved that TH1‐related interleukins IL‐2, IL‐2R and IL‐12, as well as chemokines CXCL9 and CXCL10 were increased and related to disease activity.^44^ Similar sized cohorts reported upregulated levels of chemokines CXCL9, CXCL10 and CCL18.^42^, ^45^ Serum levels of CCL18 chemokine were the most useful to differentiate from active and inactive disease, and its respective gene expression was increased at the inflammatory borders of the LS lesions, being overall a good marker to monitor the disease.^45^ Interestingly, lectin Gal‐9, tyrosine‐protein kinase receptor, soluble vascular cell adhesion molecule was also found upregulated in serum samples from the same cohort, being also potential markers.

miRNAs serve as essential regulators of cell differentiation, proliferation and survival. The involvement of miRNAs in the functioning and regulation of the skin cells and skin diseases is a rapidly advancing area in dermatological research.^53^ miRNAs have been identified to play a key role in the pathogenesis, diagnosis, and treatment of the skin diseases. It remains unknown how general miRNA regulators are, and if there are specific regulators for LS. Considering that miRNAs regulate specific pathways, specificity of miRNA to LS would be a result of having identified such a specific regulatory pathway.

To date, miRNAs have been identified to demonstrate significant effects in diverse skin inflammatory conditions such as wounds, cancer, psoriasis, scleroderma, dermatomyositis, for example, reviewed in Singhvi et al.^53^ miRNA‐29, miRNA‐21 and miRNA‐ 483‐5p were investigated in several skin conditions, mainly in SSc.^54^, ^55^, ^56^, ^57^ These miRNAs were also found to be upregulated in LS study. miRNA‐7 and miRNA‐196, both related to collagen expression, have also been studied and found downregulated in LS patients, being a potential marker for LS.^7^, ^58^ Using 38 samples of LS patients and matched controls, it was shown that the serum levels of multiple miRNAs, that is, miRNA‐181b‐5p, miRNA‐223‐3p, miRNA‐21‐5p, let 7i‐5p, miRNA‐29a‐3p and miRNA‐210‐3p were significantly increased in the LS patients compared to the healthy control (50). The level of let‐7i in the female LS patients correlated negatively with disease activity scores body surface area and modified Localised Skin Severity Index (mLoSSI). Moreover, the female patients with inactive LS had significantly higher level of let‐7i in comparison to those with active disease. The exact role of those miRNA molecules has not been revealed in LS and long‐term longitudinal research is pivotal to confirm their prognostic value.

### 2.2 Biomarkers enabling distinguishing localised scleroderma from the main differentials

It is important to differentiate LS from other conditions with similar skin manifestations to ensure accurate diagnosis and appropriate treatment. Some key skin conditions for differentiation from LS include SSc, Lichen Sclerosus, Eosinophilic Fasciitis, Morpheaform Basal Cell Carcinoma, Scleromyxedema and Localised Sclerodermoid Chronic Graft‐versus‐Host Disease (cGVHD).^59^ Today, accurate diagnosis involves a thorough clinical evaluation, medical history review, skin biopsy, histopathological examination, and may sometimes include additional diagnostic tests or imaging studies.

The main differential for LS is with no doubt SSc. A recent study found MBP antibodies in the serum of 71.4% of a cohort of 70 LS patients, differing significantly from healthy controls and SSc patients, and showing relation to pain symptoms and higher disease activity.^48^

Attachment of carbohydrate is among the most common post‐translational modifications of proteins. For immunoglobulins, it affects recognition of antigens and interaction with immune cells. The recent study recruited 93 LS patients, 298 SSc patients, and 436 healthy controls, to conduct immunoglobulin proteomics assessment.^60^ N‐glycans of purified immunoglobulin G were obtained from plasma and detected by tandem mass spectrometry. The authors examined whether the IgG‐Galactose (Gal) ratio differed between different subtypes of scleroderma. The IgG‐Gal ratio was significantly higher in SSc patients (1.139 ± 0.870) than in LS patients (0.485 ± 0.280) and controls (0.395 ± 0.190). The IgG‐Gal ratio successfully distinguished SSc patients from LS and controls (area under the curve = 0.88 and 0.81, respectively). IgG‐Gal ratios were abnormal in SSc patients and were associated with disease severity. The IgG‐Gal ratio therefore shows potential as a biomarker for the diagnosis of SSc and the differentiation from LS.

Fibrosis is a common pathophysiological response of many tissues and organs subjected to chronic injury. Despite the diverse aetiology of keloid, lacaziosis and LS, the process of fibrosis is present in the pathogenesis of all these three entities beyond other individual clinical and histological distinct characteristics. Tafuri et al. report on fibrosis immunohistochemistry study in 20 skin paraffinized samples each of these three chronic cutaneous inflammatory diseases.^31^ The presence of *α*‐smooth muscle actin (α‐SMA) and vimentin cytoskeleton antigens, CD31, CD34, Ki67, p16; CD105, CD163, CD206 and FOXP3 antigens; and the central fibrotic cytokine Transforming growth factor‐β (TGF‐β) was determined by immunohistochemistry. Vimentin was overexpressed in comparison to *α*‐SMA for all three pathologies. CD31‐and CD34‐positive blood vessel endothelial cells were present throughout the reticular dermis. Just in LS, Ki67 expression was almost absent, while. P16‐positive levels were higher and observed in reticular dermis of keloidal collagen in keloids, in collagen bundles in scleroderma and in the external layers of the granulomas in lacaziosis. *α*‐actin positive cells and rarely CD34 positive cells was found mainly in keloids, possibly related to higher p16 antigen expression, which accounts for cell senescence. CD105‐positive cells were found in perivascular tissue in close contact with the adventitia in keloids and scleroderma, while, in lacaziosis, these cells were mostly observed in conjunction with collagen deposition in the external granuloma layer. Low FOXP3 expression was observed in all lesion types. Transforming growth factor‐β was exclusive to keloid and lacaziosis lesions. For all the proposed lesion types, vimentin has been suggested to be the main myofibroblast general marker of the fibrotic process, while endothelial‐to‐mesenchymal transition (EndoMT), mesenchymal stem cells and M2 macrophages may not play a role. Similar to the case described by Tafuri et al., the also fibrosis mediator Secreted Protein Acidic and Rich in Cysteine (SPARC) is present only at sites of tissue remodelling and wound repair for different diseases. Tsuruta et al. observed that SPARC was upregulated in serum of patients with sclerosis of a small cohort of 15 patients with LS, 78 patients with other kinds of sclerosis and 15 healthy controls.^46^ Even though serum levels of SPARC were upregulated in patients with all types of sclerosis, it was especially elevated in those with LS being this protein a promising and selective candidate for LS diagnostics.

Genetic aspects of LS can aid its effective differentiation as well. Mirizio et al. reported results from RNA sequencing (RNAseq) of skin biopsies from juvenile‐onset LS.^61^ LS gene signatures compared to healthy controls showed a distinct expression of an inflammatory response gene signature (IRGS) composed of interferon genes IFNγ‐, IFNα‐, and TNFα‐associated genes. Gene enrichment analysis showed that the IRGS, including interferon‐inducible chemokines such as CXCL9, CXCL10, CXCL11, and IFNγ itself, was more highly expressed in LS patients with more inflammatory lesions. The prevalence of the IFNγ signature in the lesion biopsies of active LS patients indicates that these genes reflect clinical activity parameters and may be the promoters of early inflammatory disease. Importantly for sequencing experiment strategies and sampling, the use of paraffinized skin for sequencing was proven to be an effective substitute for fresh skin by comparing gene expression profiles.

Besides genomic markers, small regulatory RNA are exciting objects of study. Among others, microRNA are powerful short RNA molecules that are responsible for regulation of gene replication, transcription, and translation. Over decades, microRNA detection has been challenged by the fact that they are short living and present in the sample only at low (<1 fM) concentrations. As a result, before 2020, one of the few nucleic acid biomarkers described in LS was let‐7a, related to several important cell pathways as DNA damage, Janus kinase/signal transducer and activator of transcription proteins (JAK/STAT) pathway, cell cycle or apoptosis. The expression of let‐7a in dermal fibroblasts in a small cohort of 7 SSc patients, 7 LS patients and 7 healthy controls showed that the levels of the miRNA were under‐regulated in LS patients, being a potential tool for differentiate LS from SSc patients.^62^

### 2.3 Prognostic biomarkers including biomarkers indicating response to treatment

When the diagnosis LS is established and the treatment has been initiated, prognostic biomarkers can offer precise follow up and personalised management plan. Research on specific biomarkers for monitoring treatment response in LS is still evolving, and there is no universally accepted biomarker for this purpose. Clinicians primarily assess treatment response through regular clinical examinations, monitoring changes in skin thickness, texture, and overall disease activity. They may use validated assessment tools such as the Localised Scleroderma Skin Severity Index to quantify skin involvement and monitor response to treatment.

Skin biopsies may be performed before and after treatment to evaluate histological changes in the skin, such as collagen deposition and inflammation, which can indicate treatment response. Among others, sCD30 has been reported as relevant to monitor treatment outcome.^63^

Fibroblasts are the primary cell type involved in the excessive production of collagen, leading to skin fibrosis in LS. Common markers for fibroblasts include vimentin^31^, fibroblast‐specific protein 1,^64^ and platelet‐derived growth factor receptor beta^65^; all can be assessed using sera samples from patients and predict LS progression.

General blood biomarkers associated with inflammation, immune activation, and fibrosis (e.g., cytokines, growth factors, autoantibodies) can help assessing the disease activity and response to treatment in LS. Moreover, imaging with high‐frequency ultrasound or magnetic resonance imaging can provide information on skin changes in LS. In a clinical trial completed in 2013, the CD34 and FXIIIa were used to monitor outcome of skin treatment with flashlamp pulsed dye laser.^32^ Thirty patients with plaque morphea were treated with the laser. Sessions were performed biweekly for 6 months and led to improvement in skin condition, also upon follow‐up 12 months after the last laser treatment. An increased number of CD34‐positive cells were found in both the upper and the lower dermis, accompanied by reduced FXIIIa‐positive cells in the latter. Therefore, using the laser treatment with complementary use of CD34 and FXIIIa tests could be a potent new management plan for LS.

Several other cytokines have recently attracted attention in autoimmunity research and clinics. Interleukin‐6 (IL‐6) is a pro‐inflammatory cytokine that plays a significant role in immune regulation and inflammation. It is involved in various autoimmune and inflammatory conditions, including LS. Soluble IL‐6 (sIL‐6) refers to the form of IL‐6 that is found in the bloodstream and is measurable through blood tests. In LS, serum sIL‐6 levels may be elevated, reflecting an active inflammatory process. However, research regarding sIL‐6 specifically in LS is still evolving, and there may not yet be a standardized or widely accepted reference range for sIL‐6 levels in this condition. Recent mechanistic studies supported a model whereby IL‐6 trans‐signalling driven by CD4 T cell‐derived soluble IL‐6 receptor complexed with fibroblast‐derived IL‐6 promoted excess extracellular matrix gene expression.^66^ MISTRG6 mice transplanted with scleroderma skin demonstrated multiple fibrotic responses centred around human IL‐6 signalling, which was improved by the presence of healthy bone marrow‐derived immune cells. These results highlight the importance of IL‐6 trans‐signalling in pathogenesis of scleroderma and the ability of healthy bone marrow‐derived immune cells to mitigate disease.^66^ Hence, monitoring serum sIL‐6 levels may have diagnostic and prognostic implications in LS, helping to assess disease activity and predict disease progression. Targeting IL‐6 or its receptor with specific therapies (e.g., tocilizumab) is being explored in various autoimmune conditions, and it may have potential applications in LS as well.

Similarly, the specific role and levels of soluble IL‐2 receptor (sIL‐2) in LS have not been extensively studied, and there may not yet be established reference ranges or widely accepted biomarker status for sIL‐2 in this condition. Nevertheless, IL‐2 is a critical cytokine involved in the activation and proliferation of T cells. Elevated levels of sIL‐2 could suggest increased T‐cell activity in LS, potentially contributing to the inflammatory response.^67^ In a 2018 study, the connection between soluble endothelial leukocyte adhesion molecule‐1 (sE‐selectin) and sIL‐2R and the severity of skin lesions in various subtypes of LS was studied. Evaluation of disease severity, the location of skin lesions, the duration of symptoms and disease activity were assessed in relation to different LS types (generalised, plaque and linear) in patients with LS. The study included 42 patients with LS and 41 healthy subjects. Significantly higher serum levels of sE‐selectin and sIL‐2 were observed in the LS study group when compared with the control group (*p* < 0.001). The highest concentrations of sE‐selectin and sIL‐2 were observed in patients with the generalised subtype of LS. A positive, statistically significant, curvilinear relationship was shown amid the mLoSSI and sE‐selectin and sIL‐2 concentrations in the LS study group.^67^

Tumour necrosis factor‐alpha (TNF‐α) is a pro‐inflammatory cytokine that plays a crucial role in various immune and inflammatory responses. It is produced by a variety of immune cells and is known to be involved in the pathogenesis of several autoimmune and inflammatory conditions. In the context of LS (morphea), research has indicated the potential involvement of TNF‐α in the disease process.^42^ Elevated levels of TNF‐α in LS may indicate an active inflammatory process within the affected skin. Tumour necrosis factor‐alpha can stimulate the production of collagen and other extracellular matrix components. In LS, increased TNF‐α levels may contribute to the excessive collagen deposition and fibrosis seen in affected skin. Given its role in inflammation and fibrosis, TNF‐α has been explored as a potential target for therapeutic interventions in LS. Tumour necrosis factor‐alpha inhibitors (e.g., infliximab, etanercept) have been studied in some cases to assess their effectiveness in managing LS.^68^, ^69^

Together, recent data on cytokines sIL‐6, sIL‐2 and TNF‐α aligns with previous recognition of pro‐inflammatory state in LS. It is though exciting new aspect that these cytokines can become treatment targets and prognostic markers in LS.^42^, ^66^, ^67^, ^68^, ^69^

Overall, assessment of treatment response in LS is often multifaceted, involving a combination of clinical, imaging, histopathological, and patient‐reported measures. Future research is expected to shed light on specific biomarkers that can reliably predict and monitor treatment response, leading to more personalised and effective treatment strategies for individuals with LS.

Genomic biomarkers are a large field that also holds a potential to provide with prognostic biomarkers for LS. Recently, genetic signature in skin biopsies from LS patients has been analysed with next‐generation sequencing technologies. Saracino et al. performed a study on 16 LS patients with epidermal whole genome sequencing protocol.^70^ No single affected gene or single nucleotide variant has been found. However, many potential disease‐relevant pathogenic variants were present, including ADAMTSL1 and ADAMTS16. A highly proliferative, inflammatory and profibrotic epidermis profile was seen, with significantly overexpressed TNFα‐via‐NFkB, TGFβ, IL6/JAKSTAT and IFN‐signalling, apoptosis, p53 and Kirsten rat sarcoma virus (KRAS)‐responses. This is a highly diverse group of genes and pathways. Noteworthy, affected KRAS might link LS genetics to skin cancer.^71^ The authors also highlight that upregulated Interferon Alpha Inducible Protein 27 and downregulated (Laminin Subunit Alpha 4) LAMA4 potentially represent initiating epidermal ‘damage’ signals and enhanced epidermal‐dermal communication. Localised scleroderma dermis exhibited significant profibrotic, B‐cell and IFN‐signatures, and upregulated morphogenic patterning pathways such as Wnt (being this one used to induce fibrosis in scleroderma models in mice^72^). Overall, the study supports the absence of somatic epidermal mosaicism in LS, and identifies potential disease‐driving epidermal mechanisms, epidermal‐dermal interactions, and disease‐specific dermal differential‐gene‐expression in LS.

**FIGURE 1:**
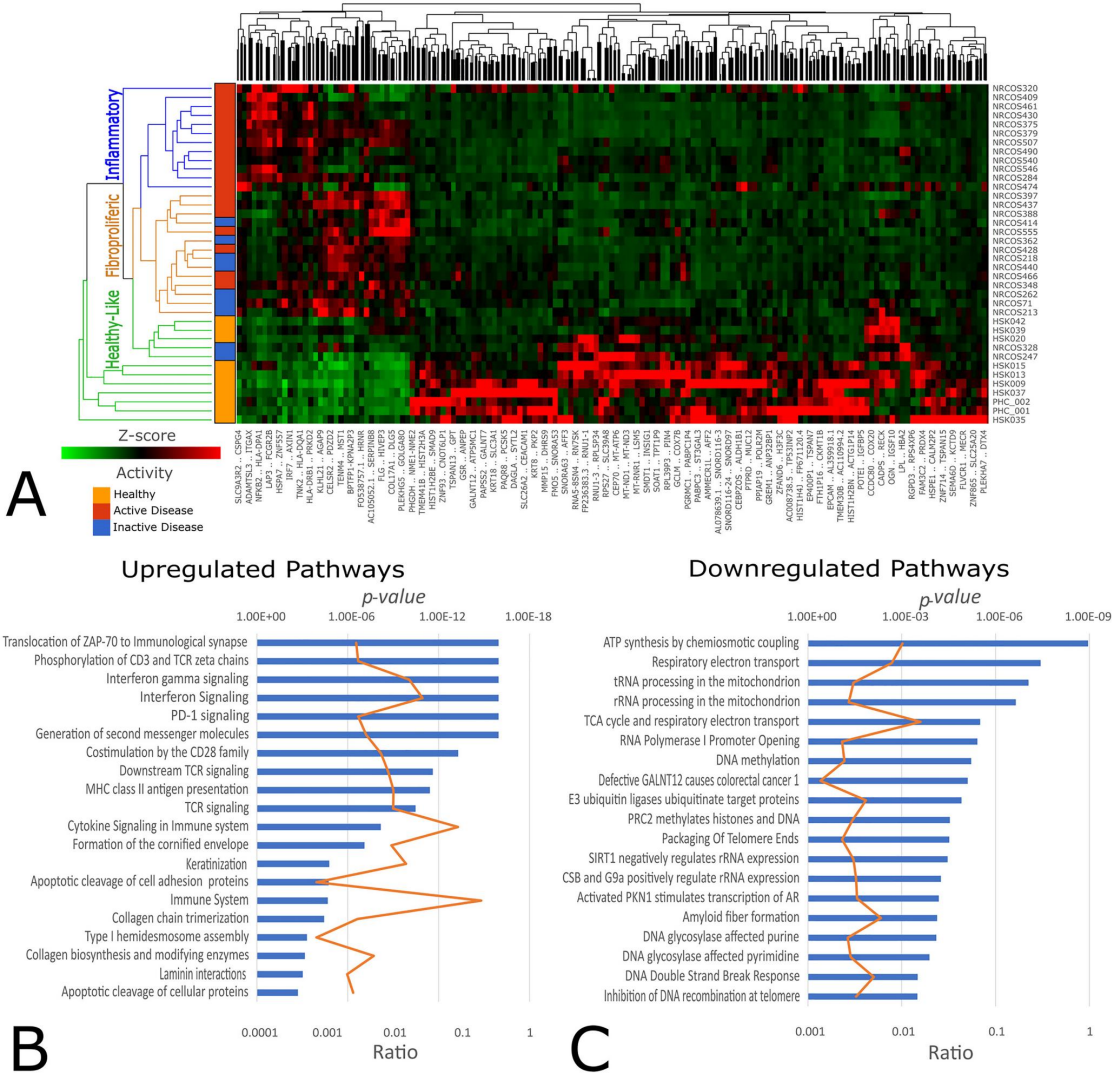
RNA transcriptome expression analyses of the skin of children with localised scleroderma (LS). (a) Dendrograms from hierarchical cluster mapping using complete linkage Euclidean distance show the groups of children with LS identified based on gene expression (genes listed on bottom) and skin histopathologic features (unique clusters designated as inflammatory, fibroproliferative, or healthy‐like), stratified by clinical disease activity status. Numbers to the right of the dendrograms represent individual skin biopsy samples. Map clustering confirmed distinct differences in juvenile LS patients compared to healthy controls. B and C, Results of pathway analyses show the functional pathways for genes that were up‐regulated (b) and those that were down‐regulated (c) in the skin of children with juvenile LS relative to healthy controls. Horizontal blue bars show the *p* values on a logarithmic scale. Vertical orange lines represent the ratio of genes listed to the number of genes associated with each pathway. AR, androgen receptor; CSB, Cockayne syndrome complementation group B protein; GALNT12, N‐acetylgalactosaminyltransferase 12; MHC, major histocompatibility complex; PD‐1, programed death 1; PKN1, serine/threonine protein kinase N1; PRC2, polycomb‐repressive complex 2; rRNA, ribosomal RNA; SIRT1 sirtuin 1; TCA, tricarboxylic acid; TCR, T cell receptor; tRNA, transfer RNA.^18^

Next, Schutt et al. analysed skin transcriptome in skin biopsy tissues from children with juvenile LS compared to paediatric healthy controls.^18^ In this study, differentially expressed genes (DEGs) were assessed for correlations with histopathologic and clinical features in children with juvenile LS and were used to group the children into distinct genetic clusters based on immunophenotype. RNA‐Seq was performed on sections of paraffin‐embedded skin tissue obtained from 28 children with juvenile LS and 10 paediatric healthy controls. As a result, 589 significant DEGs were identified in children with juvenile LS as compared to healthy controls. Hierarchical clustering was used to demonstrate 3 distinct juvenile LS immunophenotype clusters (Figure 1a). In one cluster, inflammation‐related pathways were up‐regulated, corresponding to the histologic skin inflammation score. In the second cluster, fibrosis‐related pathways were up‐regulated. In the third cluster, gene expression in the skin corresponded to the patterns seen in healthy controls. Up‐regulation of HLA class II genes was observed within the first cluster (characterised by predominant inflammation) (Figure 1b), a feature that has also been observed in the peripheral blood of patients with morphea and in the skin of patients with SSc. Moreover, the histologic scores of skin inflammation (based on numbers and categories of inflammatory cell infiltrates) were significantly correlated with the expression levels of HLA‐DPB1, HLA‐DQA2, HLA‐DRA, and STAT1 genes. Collagen thickness correlated with the expression levels of collagen organization genes as well as with genes found to be correlated with the severity of inflammation, including genes for major histocompatibility complex (MHC) class I, MHC class II, and IFNγ signalling. Identifying 3 distinct genetic signatures and associated genes is a significant step forward in terms of diagnosing and differentiating LS from SSc.

As it can already be seen in the work of Schutt et al. (Figure 1c) with the downregulation of DNA methylation pathway in LS patients,^18^ at the level of DNA genome, epigenetic regulation is another exciting field of study that also includes new findings for LS. Coit et al. studied DNA methylation differences and similarities between juvenile systemic sclerosis and juvenile LS compared to matched healthy controls.^73^ Genome‐wide DNA methylation changes in peripheral blood mononuclear cell samples were assessed using the MethylationEPIC array followed by bioinformatic analysis and limited functional assessment. A total of 105 and 144 differentially methylated sites were identified compared to healthy controls in juvenile systemic sclerosis and juvenile LS, respectively. Most differentially methylated sites and genes represented were unique to either juvenile systemic sclerosis or juvenile LS suggesting a different underlying epigenetic pattern in both diseases. Among shared differentially methylated genes, methylation levels in a CpG site in FGFR2 can distinguish between LS and healthy PBMCs with a high accuracy. Canonical pathway analysis revealed that inflammatory pathways were enriched in genes differentially methylated in SSc, including STAT3, NF‐κB, and IL‐15 pathways. In contrast, the Hippo signalling pathway was enriched in LS. This study revealed important insights into juvenile‐onset LS and suggested a potentially novel epigenetic diagnostic biomarker for LS.

Single‐cell genomics is the study of the individuality of cells with a sequencing technique. Although young, the field has now entered a more mature stage and is beginning to provide with valuable new clues on healthy immune system and on autoimmune diseases. Especially for highly heterogeneous diseases, such as LS, single cell techniques can truly contribute to in‐depth understanding of the disease pathology, underlying genetic signatures and their dynamic revelation in the disease phenotypes. This can become a path to discover new prognostic biomarkers including those indicating and even predicting response to treatment, as it already happened in oncology field. In 2020, Mirizio et al. conducted a pilot single‐cell RNAseq on paired skin biopsy specimens from 3 patients with LS, exploring different sample preparation strategies for 10× Genomics sequencing.^74^ Levels of cell viability and yield were comparable between frozen and freshly preserved cells. Furthermore, gene expression between preservation methods was collectively significantly correlated and conserved across all 18 identified cell cluster populations. The average expression of genes for major cell groups, such as keratinocytes, T/NK cells, DC/macrophages, fibroblasts, and pericytes, demonstrated a strong correlation between the average counts for each gene across all cells in the respective group. This suggests that employing standardized cryopreservation protocols for the skin tissue will help facilitate multi‐site collaborations looking to identify mechanisms of disease in disorders characterised by cutaneous pathology with single cell technology.

Another regulatory RNA that has been recently studied in LS is long non‐coding RNA (lncRNA). LncRNAs are approx. Two hundred nucleotides in length and lack protein‐coding potential. Increasing evidence indicates that lncRNAs exert an irreplaceable role in disease initiation, progression, and are novel molecular biomarkers for diagnosis and prognosis of most human diseases. Furthermore, lncRNAs and the pathways they influence might represent promising therapeutic targets. Profiling of inflammatory cells in skin samples from paediatric LS was performed, aiming at lncRNA profile analysis.^75^ Among them, CD4+ T‐cells were up‐regulated. Co‐culture dermal fibroblasts with CD4+ T‐cells promoted fibrosis of fibroblasts. Candidate lncRNAs were further explored by lncRNAs‐seq between the normal skin tissues and paediatric LS tissues, and the lncRNAs‐seq between fibroblasts co‐cultured with CD4+ T lymphocytes and control fibroblasts. By comparing the two datasets, the authors identified eight up‐regulated (LINC01184, BAALC‐AS1, AF165147.1, TRAM2‐AS1, MIR100HG, CHROMR, LINC00665, ZEB1‐AS1) and three down‐regulated (LINC00662, CARMN, PAX8‐AS1) lncRNAs, which were the potential lncRNAs for the phenotype of paediatric LS. The identified lncRNAs can become both valuable diagnostic markers and treatment candidates for paediatric LS.

### 2.4 Biomarkers in trials

To date, there are seven completed trials focusing on LS, from which one published their results (2018 completed, phase II, NCT02915835).^76^ As to the recruiting studies, morphea in adults and children clinical trial has been initiated (NCT01808937).^77^ With five hundred participants and completion planned in 2027, the trial will be first prospective six‐year‐long study where both adults and children with LS will be analysed longitudinally for multiple disease parameters including disease activity, other autoimmune conditions, and excitingly, for DNA signature.

Nice Hospital, France, is currently recruiting for a trial of microRNA in LS (NCT04148716).^78^ Prospective study with 18 participants will provide with a 2‐year insight into miRNA signature in LS. The team is studying the involvement of pro‐fibrotic “key” miRNAs called “FibromiRs”, including 3 miRNAs from the DNM3os locus (miR‐199a‐3p, miR‐199a‐5p and miR‐214 ‐ characterised by the host laboratory) associated with monitoring the response to TGF‐β in fibroblasts and their potential interaction with pharmacological treatments such as nintedanib and/or PPARγ agonists. The approach is part of a pilot study that can lead to a larger project after validation of the hypotheses.

## 3 CONCLUSIONS

Research in biomarkers is an extensive field in autoimmunity that constantly introduces new biomarkers and biological insights into the field. Despite the lack of a single optimal biomarker, a combination of them could be efficient to diagnose and assess the state of the disease.

In LS, the diagnostic perspective with existing biomarkers is still not as good as in other autoimmune diseases. This is due to the low specificity and sensitivity of the common biomarkers, which are often shared with other autoimmune diseases such as lupus and RA. Differentiation among sub‐types of sclerodermas and from other skin autoimmune conditions are also important tasks that require improved approaches. Lack of prognostic biomarkers including those reflecting response to treatment also attracts increasing attention in the research and clinical communities. Here recent genomic investigations are exciting contributions, with lncRNA, microRNA and epigenetic biomarkers being a potent new direction for research and clinics.

With three recruiting trials for new treatment strategies of LS (accessed November 2023), it becomes relevant to look up at genomic signature and responses to medication at critical checkpoints of the disease. Recent studies highlighted in this review suggest multiple proteomic and genetic biomarkers that could aid in‐depth investigation of LS and improve treatment strategies.

## CONFLICT OF INTEREST STATEMENT

None to declare.

## AUTHOR CONTRIBUTIONS

**Adrian Hernandez‐Bustos**: Writing – original draft (lead); Writing – review & editing (equal). **Begona Bolos**: Writing – original draft (supporting). **Kira Astakhova**: Conceptualisation (lead); Writing – original draft (equal); Writing – review & editing (lead).

## ETHICS STATEMENT

Not applicable.

## Data Availability

Data sharing is not applicable to this article as no new data were created or analyzed in this study.
